# Reply to ‘Dissimilarity measures affected by richness differences yield biased delimitations of biogeographic realms’

**DOI:** 10.1038/s41467-018-07252-4

**Published:** 2018-11-30

**Authors:** Mark J. Costello, Peter Tsai, Alan Kwok Lun Cheung, Zeenatul Basher, Chhaya Chaudhary

**Affiliations:** 1 Institute of Marine Science, University of Auckland, Auckland, 1142 New Zealand; 2 Bioinformatics Institute, University of Auckland, Auckland, 1142 New Zealand; 3 School of Environment, University of Auckland, Auckland, 1142 New Zealand

## Abstract

Recently, we classified the oceans into 30 biogeographic realms based on species’ endemicity. Castro-Insua et al. criticize the choices of dissimilarity coefficients and clustering approaches used in our paper, and reanalyse the data using alternative techniques. Here, we explain how the approaches used in our original paper yield results in line with existing biogeographical knowledge and are robust to alternative methods of analysis. We also repeat the analysis using several similarity coefficients and clustering algorithms, and a neural network theory method. Although each combination of methods produces outputs differing in detail, the overall pattern of realms is similar. The coarse nature of the present boundaries of the realms reflects the limited field data but may be improved with additional data and mapping to environmental variables.

## Introduction

Recognising the data limitations for the ocean^1–3^, an ideal biogeographic mapping would combine both empirical field data with expert knowledge of species ecology to account for sampling bias, and consider the validity of its findings to prior work, e.g., refs ^4–6^. We used a world data set of 65,000 marine species mapped to 5° latitude-longitude cells to map realms based on species endemicity^1^. Our primary presentation of the results used Jaccard’s coefficient of similarity, by far the most widely used index in biogeography studies^7^, and Group Average linkage clustering methods. However, we also showed similar biogeographic patterns were obtained using equal area hexagons of different sizes, and alternative coefficients, including Simpson’s coefficient that we noted is not biased by species richness (see Fig. 3 in ref. ^1^). During preliminary analyses, we had explored numerous other coefficients and clustering methods, but either found them unsuitable or did not find biologically meaningful differences in the results. The three main steps in our analysis were statistical cluster analysis of a carefully curated data set, manual exclusion of geographically isolated cells surrounded by a more biogeographically coherent cluster, and comparison of the recommended realms to previously proposed classifications (see Supplementary Table 4 in ref. ^1^). The data consisted of diverse marine species sampled by a wide variety of methods, from human observations in the field, to plankton nets, fishery trawls, and analysis of sediment samples. Thus variation in clusters could result from neighbouring cells appearing different because one had only benthic and another only pelagic samples for example, or distant cells appearing the same because their records were limited to widespread pelagic species.

Castro-Insua et al.^2^ re-analysed our data using several similarity coefficients, clustering algorithms and groupings of clusters. Their maps look like our map of the clusters before demarcation of the realms (see Supplementary Fig. 3 in ref. ^1^). This map had over 200 potential clusters but we manually merged isolated clusters (e.g. only a single 5° cell) into their neighbours because we did not consider them biogeographically robust and likely a reflection of sampling bias. We further examined the number of clusters at 1% similarity steps in the dendrogram until clusters showed no clear geographic relationship. This process was conservative and emphasised geographic coherency as a principle of the realms.

**Fig. 1:**
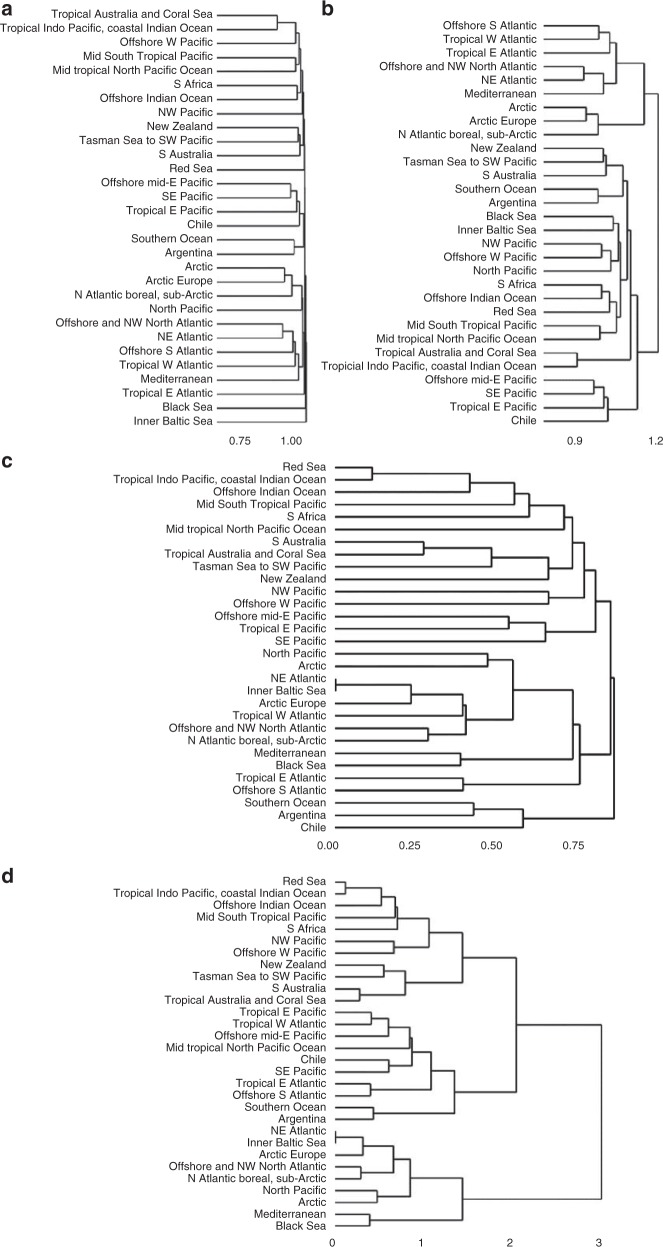
Examples of analysis of hierarchical relationships between the realms using Jaccard’s similarity coefficient with **a** Group Average and **b** Ward’s clustering methods; and Simpson’s similarity coefficient clustered using **c** Group Average and **d** Ward’s clustering methods

As suggested by Castro-Insua et al.^2^, we here compare how the use of Jaccard’s and Simpson’s coefficients, and Group Average and Ward’s clustering methods, affect the hierarchical clustering of the realms. Indeed, differences in the cluster dendrograms do occur (Fig. 1). Jaccard’s with Group Average singles out the Baltic Sea first from all other realms, and then the Black Sea (Fig. 1). Both ‘seas’ have low and variable salinity regions and thus freshwater species are present which distinguish them from all other realms. However, Jaccard’s with Ward’s coefficient places both seas’ together and within a Pacific-Indian-Southern Ocean cluster which makes no biogeographic sense given the geographic distance between these locations. In contrast, Jaccard’s with Group Average subsequently separates an Atlantic–Arctic (including part of the very North Pacific) group from a southern hemisphere group (including the Pacific, Southern and Indian Oceans). When we examine the dendrograms using Simpson’s coefficient we find Ward’s methods provide more biogeographically meaningful arrangements of the realms than using Group Average clustering (Fig. 1). In all cases with Simpson’s, the Black Sea now clusters most closely with its Mediterranean neighbour, and the Baltic Sea with its neighbouring North-East Atlantic realm. This result also makes biogeographic sense, indicating how each coefficient differently emphasises species similarities and richness. In the case of Simpson’s, we found that Ward’s provides a more balanced hierarchy than Group Average, as suggested by ref. ^8^, and it first separates the realms into two groups, a northern Atlantic-Pacific + Arctic, and mid and southern Pacific + Southern + Indian Ocean, clusters (Fig. 1).

**Fig. 2:**
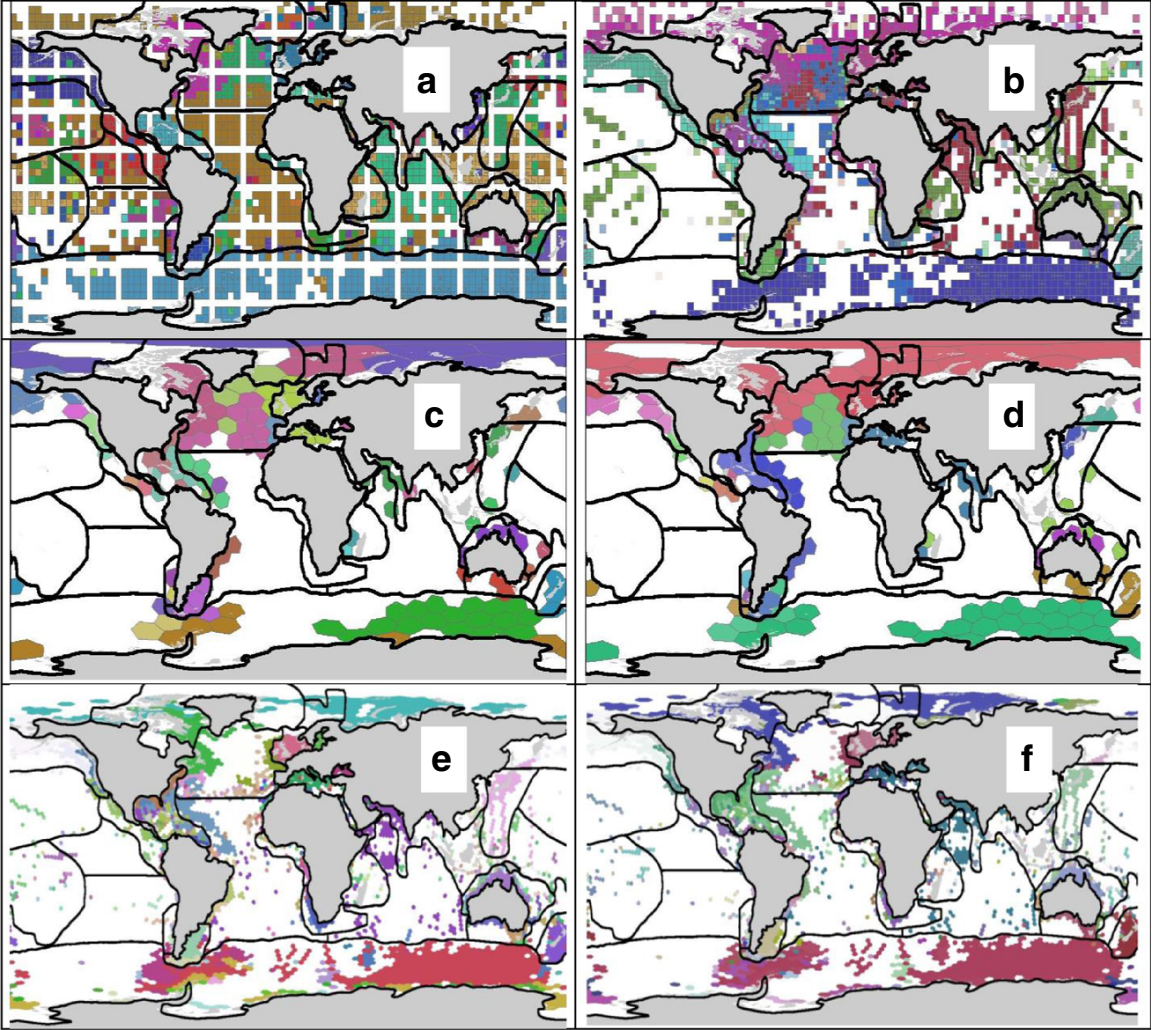
Maps of the realms overlaid on results of biogeographic analyses. The biogeographic realms are denoted with black lines. Top row is using InfoMaps on the **a** 2009 (*n* = 65,000 species, 107 clusters) and **b** 2015 (*n* = 51,670 species, 140 clusters) data from OBIS. Results of analysis of the OBIS 2015 data is shown for two sizes of hexagons (600,000 - 800,000 km^2^ and ~50,000 km^2^) using **c** Sorenson’s (38 clusters, 0.8 similarity cutoff), **d** Simpson’s (43 clusters, 0.8 cutoff), **e** Sorenson’s (252 clusters, 0.86 cut off), and **f** Simpson’s (252 clusters 0.75 cut off), similarity coefficients. The geographic gaps in **b**–**f** are because only cells with >50 samples (data with same time and place and one or more species) were used for analysis of the OBIS 2015 data. Colours were automatically assigned by the software to show cells belonging to the same biogeographic group, and are not comparable across maps

A very different method for mapping endemicity, Infomaps, has also been developed recently using neural network theory^8,9^. In contrast to the above methods, it uses each species record (latitude-longitude) directly. We also used this method (see Fig. 3 in ref. ^1^) and while similar to our proposed realms overall, it did extend the Caribbean realm southwards along the coast of Brazil. Here we map results of cluster analyses using both Sorenson’s (similar to Jaccard’s) and Simpson’s coefficients with Group Average clustering, and Infomaps on both 2009 and a 2015 OBIS data set (used in^10^ (Fig. 2). In contrast to our previous analysis, which included all 5° cells with any data, when analysing the 2015 data, we excluded cells with fewer than 50 samples. These data exclusion explains the empty hexagon cells on the map in Fig. 2. While all maps show the same general distribution of realms, they also show how the realm boundaries need better resolution.

We find both Jaccard’s and Sorenson’s with Group Average clustering, and Simpson’s with Ward’s clustering, provide biogeographically informative outcomes, as does Infomaps. Cluster analysis is exploratory and results need to be interpreted based on understanding the underlying data to judge if clusters form meaningful groups. Our realms were defined by using both these statistical methods and knowledge of the species composition of the underlying data to consider sampling bias, and then compared with prior expert classifications (see Fig. 2 and Supplementary Table 4 in ref. ^1^). The analytical alternatives suggested by Castro-Insua et al. are part of a suite of alternative methods of cluster analysis. The results of all of these methods need to be interpreted in the context of the taxonomic and geographic limitations of the underlying data, and in biogeography, in terms of geographic coherence and related knowledge.

The results of mapping the marine realms supported theories that pelagic, deep-sea and microscopic species are more widespread than benthic, coastal, and macroscopic, respectively^11^. Species’ endemicity was higher along continental shelves than in the open ocean and deep-sea. These shelves provide most fisheries and suffer most human impact, and are a focus of conservation efforts. The map provides a practical resource for conservation and resource management, and a hypothesis, which should be tested with additional data and software tools. Indeed, independent data analyses support our classification for shallow-water ostracods^12^ and have split Australia into the same northern tropical and southern temperate realms as in our analysis^13^. We show and agree with Castro-Insua et al. that alternative statistical methods can affect the hierarchical relationships of the realms, and that biogeographic research needs to consider both the similarity coefficient, clustering algorithm, and number of clusters. However, we caution against using only one method because alternatives, including Jaccard’s, Simpson’s, Infomaps, combined with alternative clustering methods, may provide new insights into biogeographic patterns. Further, these patterns must somehow reflect environmental conditions, and such data are increasingly available in the four-dimensions of latitude, longitude, depth, and time^14,15^.

## Data Availability

The primary data used here are freely available from OBIS (https://www.iobis.org). The aggregated species by 5° cell matrix finally used in the data analysis is available from Figshare at https://figshare.com/s/e11b3f7769ef353c6262 and 10.17608/k6.auckland.5086654

## References

[1] Costello, M. J. et al. Marine biogeographic realms and species endemicity. *Nat. Commun*. **8**, 1057 (2017). 10.1038/s41467-017-01121-2 PMC5648874 29051522

[2] Castro-Insua, C. A., Gomez-Rodriguez, C., Baselga, A. Dissimilarity measures affected by richness differences yield biased delimitations of biogeographic realms. *Nat*. *Commun*. 10.1038/s41467-018-06291-1 (2018). 10.1038/s41467-018-06291-1 PMC6269499 30504812

[3] Costello MJ, Vanhoorne B, Appeltans W (2015). Conservation of biodiversity through taxonomy, data publication and collaborative infrastructures. Conserv. Bio. l.

[4] Spalding MD (2007). Marine ecoregions of the world: a bioregionalization of coastal and shelf areas. BioScience.

[5] Ekman, S. *Zoogeography of the Sea* 417 (Sidgwick and Jackson, London, 1953).

[6] Spalding MD, Agostini VN, Rice J, Grant SM (2012). Pelagic provinces of the world: a biogeographic classification of the world’s surface pelagic waters. Ocean Coast. Manag..

[7] Kreft H, Jetz W (2010). A framework for delineating biogeographical regions based on species distributions. J. Biogeog..

[8] Edler D, Guedes T, Zizka A, Rosvall M, Antonelli A (2016). Infomap Bioregions: Interactive mapping of biogeographical regions from species distributions. Syst. Biol..

[9] Vilhena DA, Antonelli A (2015). A network approach for identifying and delimiting biogeographical regions. Nat. Comm..

[10] Chaudhary C, Saeedi H, Costello MJ (2017). Marine species richness is bimodal with latitude: a reply to Fernandez and Marques. Trends Ecol. Evol..

[11] Costello MJ, Chaudhary C (2017). Marine biodiversity, biogeography, deep-sea gradients, and conservation. Curr. Biol..

[12] Yasuhara, M. et al. Eocene shallow-marine ostracods from Madagascar: southern end of the Tethys? *J. Syst. Palaeontol*, 1–53, 10.1080/14772019.2018.1453555 (2018).

[13] Cresswell AK (2017). Translating local benthic community structure to national biogenic reef habitat types. Glob. Ecol. Biogeogr..

[14] Sayre RG (2017). A three-dimensional mapping of the ocean based on environmental data. Oceanography.

[15] Costello MJ, Basher Z, Sayre R, Breyer S, Wright D (2018). Stratifying ocean sampling globally and with depth to account for environmental variability. Sci. Rep..

